# Hypoxia, acidification and oxidative stress in cells cultured at large distances from an oxygen source

**DOI:** 10.1038/s41598-022-26205-y

**Published:** 2022-12-15

**Authors:** Natali D’Aiuto, Jimena Hochmann, Magdalena Millán, Andrés Di Paolo, Ronell Bologna-Molina, José Sotelo Silveira, Miguel Arocena

**Affiliations:** 1 Cátedra de Bioquímica y Biofísica, Facultad de Odontología, Universidad de la República, Montevideo, Uruguay; 2 Departamento de Genómica, Instituto de Investigaciones Biológicas Clemente Estable, Montevideo, Uruguay; 3 Departamento de Fisiología, Facultad de Medicina, Universidad de la República, Montevideo, Uruguay; 4 Departamento de Patología Molecular, Facultad de Odontología, Universidad de la República, Montevideo, Uruguay; 5 Sección Biología Celular, Facultad de Ciencias, Universidad de la República, Montevideo, Uruguay

**Keywords:** Biochemistry, Cancer, Cell biology

## Abstract

Hypoxia is a condition frequently encountered by cells in tissues, whether as a normal feature of their microenvironment or subsequent to deregulated growth. Hypoxia can lead to acidification and increased oxidative stress, with profound consequences for cell physiology and tumorigenesis. Therefore, the interplay between hypoxia and oxidative stress is an important aspect for understanding the effects of hypoxic microenvironments on cells. We have used a previously developed variant of the method of coverslip-induced hypoxia to study the process of acidification in a hypoxic microenvironment and to simultaneously visualize intracellular levels of hypoxia and oxidative stress. We observed high accumulation of CO_2_ in hypoxic conditions, which we show is the main contributor to acidification in our model. Also, increased levels of oxidative stress were observed in moderately hypoxic cells close to the oxygen source, where the mitochondrial membrane potential was preserved. Conversely, cells at large distances from the oxygen source showed higher levels of hypoxia, milder oxidative stress and reduced mitochondrial membrane potential. Our results contribute to characterize the interplay between reduced oxygen levels, acidification and oxidative stress in a simple in vitro setting, which can be used to model cell responses to an altered environment, such as the early tumor microenvironment.

## Introduction

Cells have evolved sophisticated mechanisms for sensing and adapting to low levels of O_2_, which are frequently encountered in tissues, as well as during early tumor development^1,2^. Cellular responses to hypoxia are centered on the action of hypoxia inducible factors (HIF), requiring stabilization of the HIF-1 subunit HIF-1α, which, together with HIF-1β, form a functional HIF-1 heterodimer that activates the transcription of multiple target genes^3^. Increased levels of HIF-1α are associated with hypoxia in tissues and cells, and are commonly detected by immunostaining^4^. A more direct method for hypoxia assessment by immunostaining is the use of nitroimidazoles such as pimonidazole, which under low intracellular levels of O_2_ forms adducts with cellular proteins, detectable by specific antibodies^5^. More recently, hypoxia specific fluorescent probes have become available, allowing for real time imaging of the hypoxic status of cells under different experimental conditions^6^.

The tumor microenvironment has been shown to have an acidic extracellular pH, which might constitute a selective pressure favoring the survival of more aggressive tumor cells, as well as a factor leading to decreased chemotherapy effectiveness^7^. Tumor acidification is thought to be related to lactate production by increased anaerobic glycolysis in hypoxic conditions, although CO_2_ production is an alternative mechanism of acidification as well^8^. For instance, tumor cells with impaired glycolysis show an acidified microenvironment, linked to their CO_2_ production^9^. Therefore, in vitro models of the tumor microenvironment that allow to determine the contributions of lactate and CO_2_ production to extracellular acidification would contribute towards understanding the mechanisms of pH decrease during tumor growth.

Hypoxic conditions are also associated with higher intracellular levels of oxidative stress, particularly in the form of increased reactive oxygen species (ROS), produced mainly at the level of the mitochondrial respiratory complex III^10^. Increased ROS as a consequence of hypoxia participate in the O_2_ sensing mechanism of cells^11^, but they can also have adverse consequences, particularly in the frequently hypoxic tumor microenvironment, where they contribute to increased tumor genetic instability and invasiveness^12,13^. Proposed mechanisms of hypoxia-induced generation of ROS include a slowed electron transport chain, which would allow partially reduced ubiquinone (ubisemiquinone) to transfer electrons to O_2_, even if O_2_ levels are low^14,15^. Both a functional electron transport chain and the maintenance of mitochondrial membrane potential have been linked to mitochondrial generation of ROS during hypoxia^16^. In particular, mitochondrial ROS production occurs as a transient burst of superoxide (O_2_^-^) generation during the first minutes of acute hypoxia, which is related to the activation of the mitochondrial Na^+^/Ca^2+^ exchanger ^17,18^.

Increased oxidative stress can be detected not only under low ambient O_2_ but also immediately after reoxygenation, suggesting a complex interplay between varying levels of microenvironment O_2_ levels and ROS production^19^. Therefore, important aspects of the study of hypoxia-induced oxidative stress are the methodologies used to recreate a hypoxic cellular environment and to evaluate intracellular hypoxiaas well as oxidative stress levels. In particular, to define more precisely the links between hypoxia and oxidative stress, methodologies devised to simultaneously assess the intracellular hypoxic and ROS status would constitute a useful contribution. Microfluidic systems have been used to expose cells to different levels of ambient O_2_ while simultaneously imaging the fluorescence intensity of ROS sensitive fluorescent probes^20^. Alternatively, cell culture chambers that restrict gas exchange with the environment have been used to create cell-generated oxygen gradients, and to image hypoxic gradients and other relevant parameters, such as mitochondrial membrane potential^21,22^.

Recently, we have modified the method of coverslip-induced hypoxia to create simple cell culture chambers where cells can be cultured at large distances from an oxygen source, generating high levels of intracellular hypoxia that are compatible with live cell imaging^23^. In the present study, we have used this method to simultaneously visualize the hypoxic and oxidative status of cells at large distances from an oxygen source.

On the other hand, in our previous study, we used the modified method of coverslip-induced hypoxia in LNCaP cells, which is a metastatic and highly glycolytic cancer cell line, and therefore represents a cellular model of advanced cancer progression^23^. However, it would also be important to use a cellular model more representative of intermediate stages of malignant transformation, considering in particular that hypoxia in the early tumor microenvironment is a powerful selective force driving tumor progression^2^.

In the present study, we have used, as a cell model, the human keratinocyte, immortalized, non-tumorigenic HaCaT cell line, transduced with viral oncogenes E5, E6 and E7 of Human Papilloma Virus type 18 (HPV-18) oncogenes, which represents a cellular model of an intermediate stage of malignant transformation induced by high risk HPV. Previous results from our group have shown that this cell line exhibited increased levels of oxidative stress as well as a more invasive phenotype compared to the HaCaT parental cell line without viral oncogenes, which are non transformed cells^24^. In both HaCaT parental and HaCaT transduced with viral oncogenes (henceforth referred as HaCaT E5/E6/E7–18), we have observed increased oxidative stress levels when cells are close to the oxygen source, observingalso mild intracellular hypoxia levels and preservation of the mitochondrial membrane potential in this situation. In cells at large distances from an oxygen source, which are highly hypoxic, oxidative stress levels are reduced and the mitochondrial membrane potential decreases. Moreover, we have observed increased acidification, accompanied by increased lactate and CO_2_ levels. Next, we have developed a simple approach to assess the contributions of lactate and CO_2_ to pH decrease, showing that, in our in vitro model of the tumor microenvironment, the main contribution to acidification comes from CO_2_ production.

Therefore, our results show that the modified method of coverslip-induced hypoxia can be used to recreate a hypoxic, acidified microenvironment. With this method, the contributions of lactate and CO_2_ to acidification can be assessed, and in particular the oxidative and hypoxic status of cells can be visualized simultaneously, providing a method to study the contributions of different environmental stresses on cell physiology.

## Results

### Intracellular hypoxia in cells at large distances from an oxygen source

**Figure 1:**
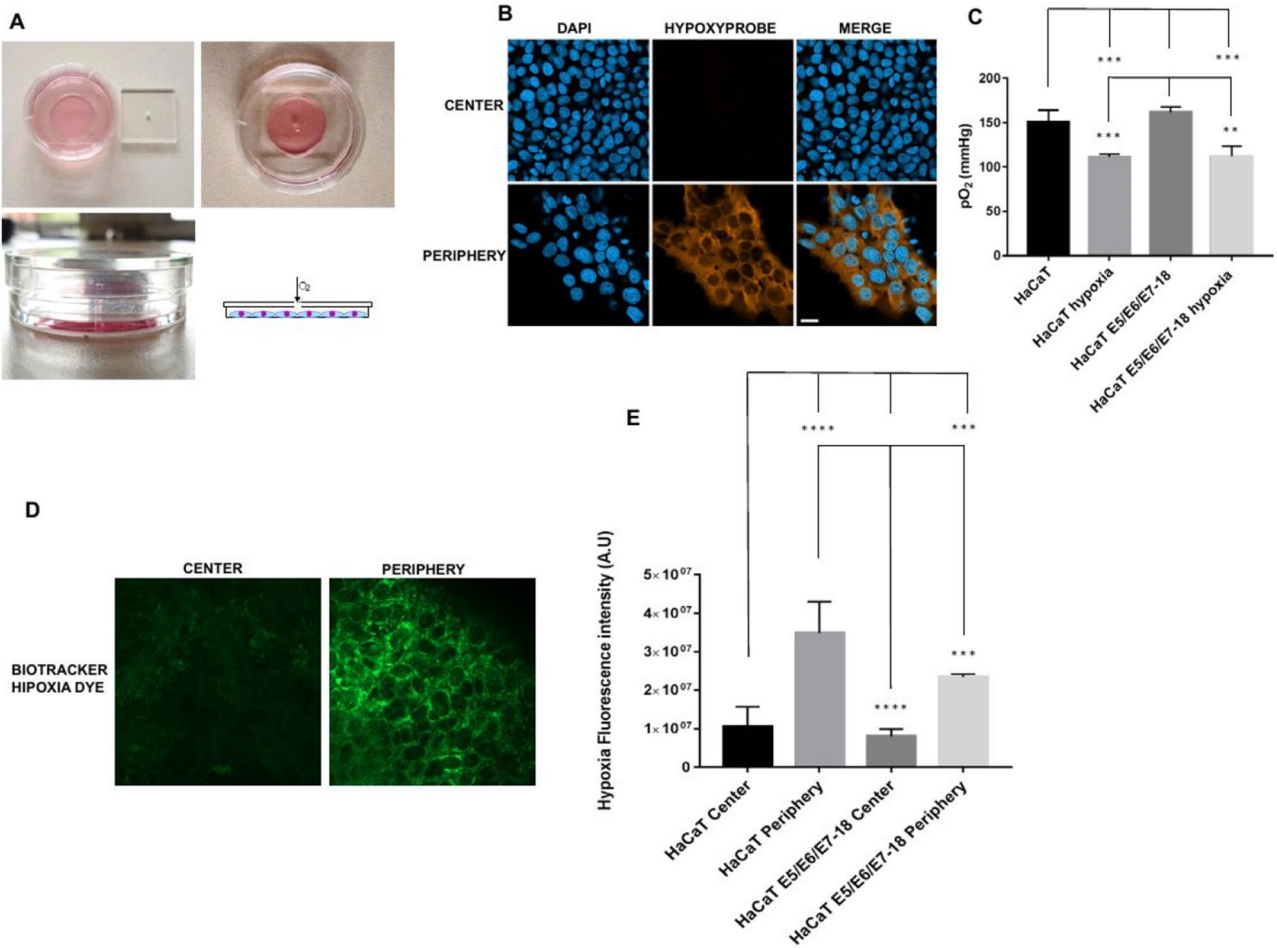
(**A**) Schematics of the cell chamber used in the coverslip-induced hypoxia variant. An acrylic coverslip with a central hole is placed on top of the well of a 35 mm glass bottom dish. The central hole is the only oxygen source, and cells inside the well are at varying distances from it. (**B**) Representative images of pimonidazole adduct detection (Hypoxyprobe), shown here in HaCaT E5/E6/E7 HPV-18 cells cultured under coverslips for 24 h. Images from the central zone or the periphery of the cell chamber are shown. (**C**) Quantification of O_2_ levels in HaCaT parental and HaCaT E5/E6/E7 HPV-18 cells in normoxia and with a coverslip. (**D**) BioTracker™ 520 Green Hypoxia Dye signal in control cells, or in cells under coverslips for 24 h, either in the central zone or the periphery of the cell chamber. (**E**) Quantification of hypoxia signal through BioTracker Green Hypoxia Dye in HaCaT E5/E6/E7–18 and parental HaCaT cells. (*) *P* < 0.05, (**) *P* < 0.01, (***) *P* < 0.001. Scale bar: 20 μm.

HaCaT cells (both parental and E5/E6/E7–18) were cultured for 24 h under a coverslip with a central hole that serves as the only oxygen source (see Fig. 1A for a schematic of the method). In the periphery of the cell chamber, cells developed the high levels of intracellular hypoxia that can be detected by pimonidazole, but not near the center of the chamber (Fig. 1B), or at intermediate regions (data not shown). Therefore, we have studied intracellular hypoxia and other cellular properties (as detailed in following sections) in cells either close to the oxygen source (cells near the center of the chamber) or at a large distance from this source (cells at the periphery of the chamber). However, when instead of pimonidazole we used BioTracker™ 520 Green Hypoxia Dye to visualize intracellular hypoxia in live cells, after 24 h of coverslip treatment a moderate level of signal was observed near the center of the chamber, although the signal was much stronger in the periphery, indicating that intracellular hypoxia levels are high in the periphery but that milder levels can also be found in the central zone of the chamber (Fig. 1D,E). O_2_ levels in the culture medium are significantly reduced after 24 h of coverslip treatment (Fig. 1C), which, taken together with the previous results, suggests that cell respiration lowers O_2_ levels in the coverslip treatment condition, and therefore cells in the periphery of the chamber, far from the oxygen source, become more hypoxic than cells in the central region of the chamber, near the oxygen source.

### Acidification, CO_2_ and lactate production after coverslip-induced hypoxia

**Figure 2:**
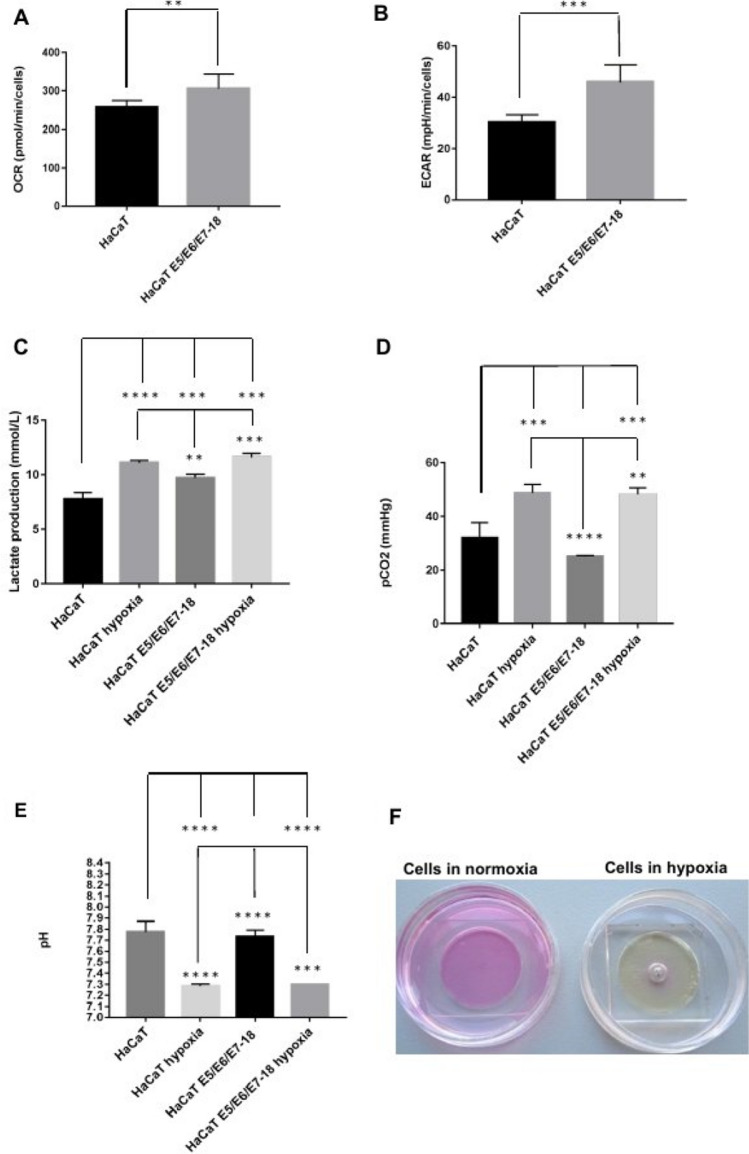
Measurement of metabolic parameters in HaCaT parental and HaCaT E5/E6/E7-18 cell lines. (**A**) Oxygen Consumption Rate (OCR) (**B**) Extracellular Acidification Rate (ECAR) (**C**) Lactate production levels measured in HaCaT parental and HaCaT E5/E6/E7–18 cells in normoxia and hypoxia conditions. (**D**) CO_2_ levels measured in HaCaT parental and HaCaT E5/E6/E7-18 cells in normoxia and hypoxia conditions. (**E**) pH measurements in HaCaT parental and HaCaT E5/E6/E7–18 cells in normoxia and hypoxia. (**F**) Changes in phenol red culture medium can be seen after coverslip treatment. (*) *P* < 0.05, (**) *P* < 0.01, (***) *P* < 0.001.

In our previous study, we observed that HaCaT E5/E6/E7–18 cells have increased expression of the glycolytic enzyme Enolase2^24^ compared to parental HaCaT cells. To further characterize metabolic differences between HaCaT parental and E5/E6/E7–18 cells in normoxic conditions, we measured oxygen consumption rate (OCR) and extracellular acidification rate (ECAR) with a Seahorse XF24 Extracellular Flux Analyzer. HaCaT E5/E6/E7–18 cells showed increased OCR and ECAR compared to HaCaT parental cell lines (Fig. 2A,B). Slightly increased levels of lactate were also observed in the cell supernatants for HaCaT E5/E6/E7–18 cells compared to HaCaT parental cells, both in normoxia and after coverslip treatment (Fig. 2C). Independently of these metabolic differences between HaCaT E5/E6/E7–18 cells and parental cells, both cell lines showed significantly increased levels of lactate and CO_2_ in the cell supernatant after coverslip treatment (Fig. 2D), whereas the pH of the cell supernatant decreased significantly after coverslip treatment for both cell lines (Fig. 2E,F). These results confirm our previous observations of extracellular acidification after coverslip treatment^23^, and suggest that both lactate and CO_2_ increase contribute to pH decrease.

### Contributions of CO_2_ and lactate to acidification after coverslip-induced hypoxia

To assess more precisely the contributions of lactate and CO_2_ to the observed pH decrease, we developed a simple approach, based on the Kassirer–Bleich approximation for the CO_2_ derived bicarbonate, and on the electrical neutrality in a medium containing abundant inorganic ions such as Na^+^, K^+^, Ca^2+^ and Cl^−^^25^. As detailed in Appendix 1, we obtain the following equation, which relates [H^+^] to [lactate] and pCO_2_:

$$\left[{H}^{+}\right]\approx \frac{0.000024 \times {pCO}_{2}}{{\Delta }_{\text{inorganic ions}}-\left[lactate\right]}$$

where Δ_inorganic ions_ is the difference in charge between the aforementioned inorganic ions. As shown in Appendix 1, this equation fits closely the experimental pH values observed, and it allows to show in a straightforward manner that the observed increase in pCO_2_ contributes much more importantly than the observed increase in lactate concentration to the observed decrease in pH. Therefore, in our model of coverslip-induced hypoxia and with the HaCaT cell lines used, the acidification we observed is related mainly to CO_2_ accumulation.

### Simultaneous visualization of hypoxia and oxidative stress in coverslip-induced hypoxia

**Figure 3:**
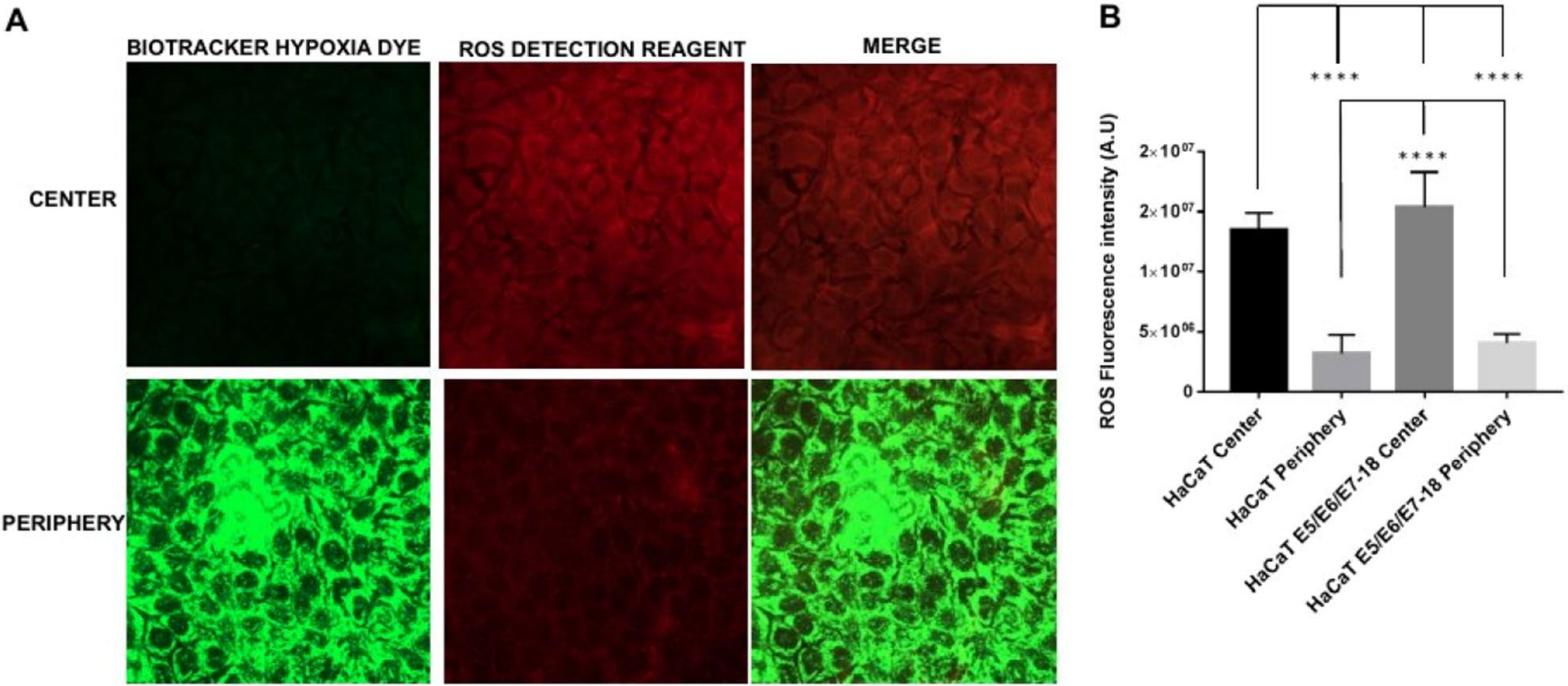
Simultaneous detection of intracellular hypoxia and ROS levels (**A**). Representative images of BioTracker™ 520 Green Hypoxia Dye, and ROS Detection Reagent Deep Red signal in cells under coverslips for 24 h, either in the central zone or the periphery of the cell chamber. (**B**) Quantification of Hypoxia and ROS intensity (mean ± SEM) in normoxia and coverslip conditions for HaCaT parental and HaCaT E5/E6/E7–18 cells.

Next, live cells under coverslips were incubated simultaneously with the BioTracker™ 520 Green Hypoxia Dye (498/520 nm excitation/emission) and with the ROS Detection Reagent Deep Red (640/675 nm excitation/emission). The ROS Detection Reagent signal was significantly higher near the center of the chamber (close to the oxygen source), where intracellular hypoxia was mild, than in the periphery (farther from the oxygen source), where intracellular hypoxia was high (Fig. 3A and B).

### Simultaneous visualization of hypoxia and mitochondrial membrane potential in coverslip-induced hypoxia

**Figure 4:**
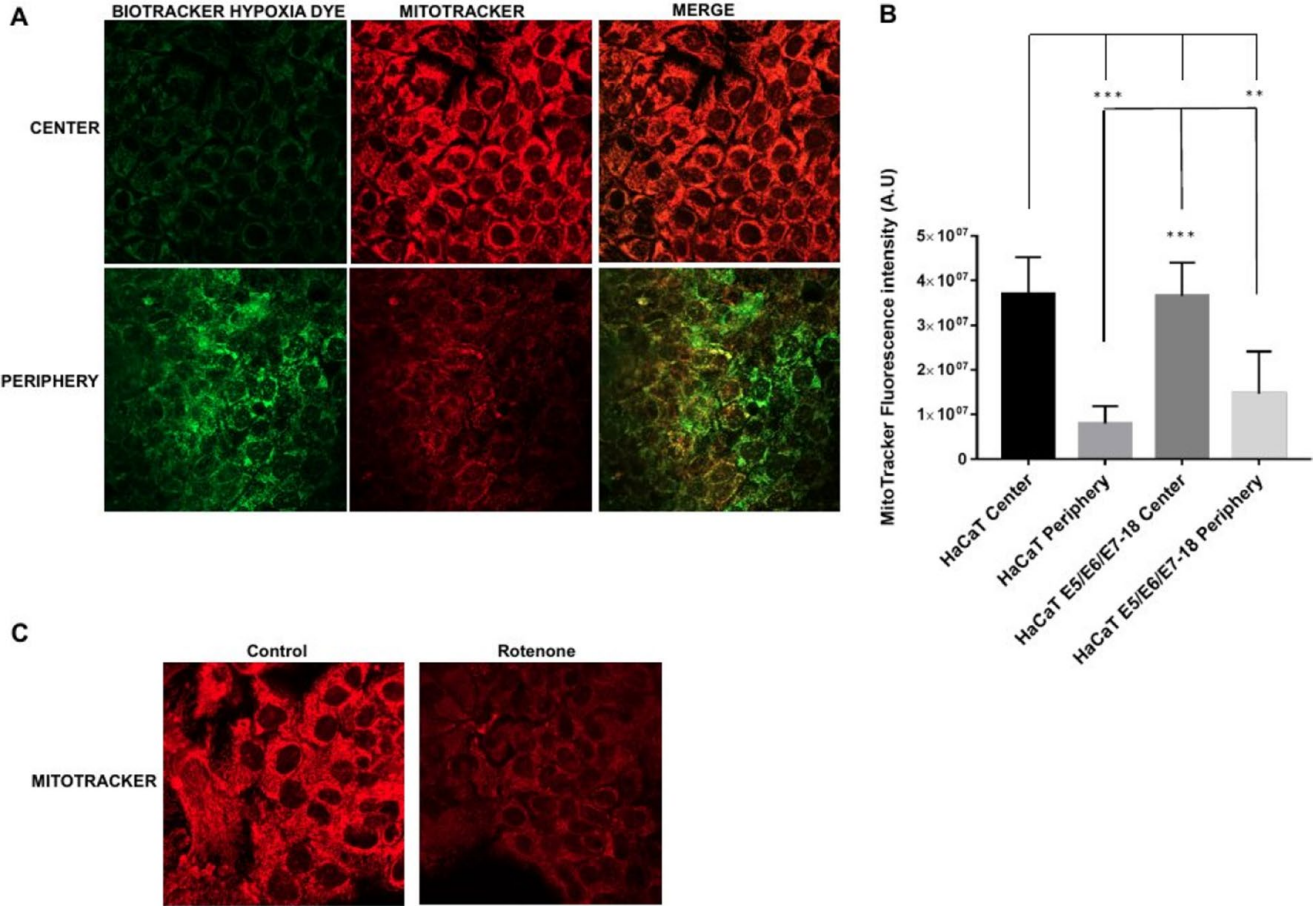
Mitochondria potential in HaCaT parental and HaCaT E5/E6/E7–18 cell lines. (**A**) Representative images of MitoTracker Deep Red FM signal in cells under coverslips for 24 h, either in the central zone or the periphery of the cell chamber. (**B**) Quantification of mitochondrial potential (mean ± SEM) in normoxia and hypoxia conditions for HaCaT parental and HaCaT E5/E6/E7–18 cells. (**C**) Representative images showing Mitotracker signal in HaCaT cells in control conditions and after 1 h rotenone (1 μM). (*) *P* < 0.05, (**) *P* < 0.01, (***) *P* < 0.001. Scale bar: 20 μm.

We then assessed the status of the mitochondrial membrane potential with the fluorescent probe Mitotracker Deep Red, that shows mitochondrial accumulation dependent upon mitochondrial membrane potential^26,27^, confirmed also by decreased staining intensity after treatments that reduce mitochondrial membrane potential^28^. Also, given that Mitotracker Deep Red has an excitation/emission maxima of 644/665 nm, we used it simultaneously with the Biotracker Green Hypoxia Dye. After 24 h under coverslips, cells showed significantly decreased Mitotracker Deep Red fluorescence intensity in the periphery, where the hypoxia signal was high, compared with the central region of the chamber, where the hypoxia signal was low (Fig. 4A and B). Moreover, a 30 min treatment with the electron transport inhibitor rotenone (1 μM) caused a decrease in fluorescence intensity as well, confirming that Mitotracker Deep Red fluorescence intensity is sensitive to the mitochondrial membrane potential (Fig. 4C). Our results therefore indicate that in the periphery of the chamber, where cells are furthest from the oxygen source and experiencing high intracellular hypoxia, mitochondrial membrane potential is significantly reduced compared to cells near the oxygen source, which experience milder levels of intracellular hypoxia.

## Discussion

In this study, we have analyzed the acidification, hypoxia and oxidative stress generated by a modified method of coverslip-induced hypoxia that permits to culture cells at large distances from an oxygen source. We observed decreased O_2_ in cell supernatants after 24 h of coverslip treatment, at which time we detected highly hypoxic cells in the periphery of the chamber, furthest from the oxygen source, both by pimonidazole staining and by using the live cell hypoxia marker BioTracker™ 520 Green Hypoxia Dye. Hypoxia detection by pimonidazole adduct formation is sensitive only to very low intracellular levels of O_2_^5^, and we did not observe pimonidazole staining in cells in the central region of the chamber, near the oxygen source. However, we detected a moderate hypoxia signal in cells in this region with the BioTracker™ 520 Green Hypoxia Dye, in agreement with the manufacturers' reported properties of the Biotracker dye of detecting milder hypoxic conditions than pimonidazole. Therefore, taken together, our results can be interpreted as indicating that O_2_ decreases after coverslip treatment due to cell respiration, and that cells farther from the oxygen source become as a consequence highly hypoxic, whereas cells near the oxygen source become only moderately hypoxic.

Coverslip treatment also leads to a significant decrease in the pH of the extracellular medium, while causing accumulation of both lactate and CO_2_. As shown in Appendix 1, we derived a simple expression for [H^+^] as a function of [lactate] and pCO_2_, using an approach drawing on the Kassirer–Bleich approximation for the CO_2_ derived bicarbonate, and on the approach pioneered by Stewart for assessing the importance of CO_2_ in the pH of body fluids, in particular the consideration of electrical neutrality in a medium containing abundant inorganic ions^25^. We obtained a very good fit between the experimental pH values and the values predicted by this expression and, in particular, using the experimental values for [lactate] and pCO_2_, it is straightforward to show that, in our experimental setting, CO_2_ accumulation is the main contributor to pH decrease after coverslip-induced hypoxia. These results are in agreement with previous studies, which show that tumor acidification is related to CO_2_ production by tumor cells, and that acidification can occur even in glycolysis impaired cells or cells deficient in lactate dehydrogenase, which have markedly decreased lactate production^9,29^. In our model, CO_2_ accumulates due to the coverslip precluding gas exchange with the environment. In the microenvironment of non-vascularized or poorly vascularized tumors, CO_2_ would be expected to accumulate, either by cellular respiration until oxygen is completely consumed, or by other metabolic routes, such as the pentose phosphate pathway^8,9^, leading to acidification. Therefore, our modified method of coverslip-induced hypoxia constitutes a simple yet useful model for analyzing the different contributions of cell metabolism towards acidification in contexts such as the tumor microenvironment.

When we visualized simultaneously hypoxia and oxidative stress, by combining the BioTracker™ 520 Green Hypoxia Dye and the ROS Detection Reagent Deep Red probe, we observed increased levels of oxidative stress in moderately hypoxic cells near the center of the chamber, whereas levels of oxidative stress in highly hypoxic cells in the periphery of the chamber were reduced. At the same time, we observed a decrease in fluorescence intensity of the MitoTracker Deep Red probe, which is sensitive to the mitochondrial membrane potential, in cells in the periphery of the chamber, where intracellular hypoxia is highest. Taken together, these results suggest that, at high levels of intracellular hypoxia, mitochondrial membrane potential decreases, leading to lower oxidative stress levels compared with cells with moderate intracellular hypoxia, in which the mitochondrial membrane potential is preserved. In agreement with these results, both a functional electron transport chain and mitochondrial membrane potential have been shown to be required for ROS production in hypoxia^16^. Moreover, the mitochondrial ROS burst that has been shown in a previous study to occur in the first minutes of hypoxia has been related to a certain degree of O_2_ availability remaining inside cells at the beginning of hypoxia^17^. The cells that are close to the oxygen source in our coverslip-induced hypoxia method would be in a similar situation to the cells at the beginning of hypoxia in that study, with a certain level of O_2_ remaining inside the cells (which in our cells translates as moderate intracellular hypoxia as visualized by the BioTracker™ 520 Green Hypoxia Dye). Therefore, the increased level of oxidative stress that we observed in cells close to the oxygen source is in agreement with the increased ROS production observed previously in cells at the initial stages of hypoxia^17^.

Our results also highlight the usefulness of our modified coverslip-induced hypoxia method for simultaneously visualizing levels of intracellular hypoxia and oxidative stress when studying the links between hypoxia and ROS production. In particular, the temporal dynamics of ROS production in hypoxia would be an important issue that could be analyzed with this method^17^. Also, it is important to consider cell type variabilities, as for instance similar extracellular O_2_ levels can result in markedly different intracellular O_2_ levels depending on the cell type, with more glycolytic cell types showing higher intracellular O_2_ levels at low extracellular levels of O_2_^30^. Therefore, simultaneously visualizing levels of intracellular hypoxia and oxidative stress would allow to assess whether differences in intracellular ROS levels across cell types might be related to cell type dependent levels of intracellular hypoxia under similar low O_2_ culture conditions.

During tumor development, tumor cells are subjected to fluctuating O_2_ levels, either in the avascular state or due to a disorganized vascular network after angiogenesis has occurred, giving rise to conditions of intermittent hypoxia, which render tumor cells more resistant to apoptotic stimuli^31^. If moderate intracellular hypoxia, rather than nearly anoxic intracellular conditions, is associated with increased intracellular ROS levels, as suggested by our results, it would be expected that tumor cells subjected to intermittent hypoxic conditions would develop milder intracellular hypoxia levels, and consequently higher intracellular ROS levels, favoring the selection of more resistant and aggressive tumor clones^12^. In addition, intermittent hypoxia could also be expected to cause increased CO_2_ accumulation, due to periods of cellular respiration followed by impaired gas exchange, therefore leading to extracellular acidification, which constitutes another selective pressure towards more aggressive tumor cells^8^.

Our modified method of coverslip-induced hypoxia allows to study both the process of acidification and the interplay between intracellular hypoxia and oxidative stress as the distance from an oxygen source increases. In particular, in this study we have used HaCaT E5/E6/E7–18 cells, a recently developed cellular model of an intermediate stage of malignant transformation induced by high risk HPV, which have increased invasion capabilities and a more glycolytic metabolism compared to parental, non transduced HaCaT cells, but are still far from developing the highly glycolytic and invasive phenotypes of cell lines derived from advanced stage cancers^24^. Using this cellular model, we have been able to assess the contributions of lactate and CO_2_ to extracellular acidification, and to simultaneously assess both the oxidative stress and hypoxic status of cells either close to an oxygen source or at large distances from it. Our results suggest that cells at intermediate stages of malignant transformation, when exposed to the hypoxic, avascular early tumor microenvironment, will cause extracellular acidification mainly through CO_2_ accumulation, and will develop higher levels of ROS in regions closer to the capillaries from the underlying stroma, which constitute the oxygen source. Therefore, in this study we have shown that our modified method of coverslip-induced hypoxia constitutes a simple yet useful model to study how an altered microenvironment can impact processes such as tumor progression.

## Materials and methods

### Cell culture

Spontaneously immortalized human keratinocyte (HaCaT) cells were purchased from Banco de células do Rio de Janeiro (BCRJ), Brazil (batch number 001071, certificate of analysis provided by the supplier) and maintained in Dulbecco’s modified eagle’s medium. (DMEM) low glucose medium (Capricorn, Ebsdorfergrund, Germany) supplemented with 10% fetal bovine serum (FBS) (Gibco, MA, USA). HaCaT cells were tested internally for mycoplasma by polymerase chain reaction (PCR). HaCaT E5/E6/E7 HPV-18 cells were obtained through co-infection with a retroviral vector carrying the MSCV-N-puro-18E5 plasmid (Addgene # 37882, MA, USA) and with a pLXSN retroviral vector that contained cloned HPV-18 E6/E7 genes gently provided by Dr. Laura Sichero. The preparation and characterization of these cell lines was detailed in Hochmann et al.^24^.

### Coverslip-induced hypoxia

To culture cells at large distances from an oxygen source, we used the variant of coverslip-induced hypoxia described previously^23^. Briefly, cells were cultured in the 10 mm radius wells of glass bottom dishes (35 mm diameter, Cellvis, CA, USA), and after cells were adhered the wells were covered with square acrylic coverslips (24 mm width, 2 mm thickness) with a square hole in the middle (1 mm width). In this way, cell culture chambers are obtained where cells in the periphery of the chamber are located at 10 mm from the coverslip hole (which serves as the only oxygen source). The method is depicted in Fig. 1A.

### Hypoxia detection in fixed cells

For hypoxia detection in fixed cells, we used a hypoxia detection kit (Hypoxyprobe, MA, USA) as described previously^23^. Cells under coverslips for 22 h were incubated with the hypoxia marker pimonidazole for another 2 h at a final concentration of 200 μM in the medium. Next, cells were fixed 10 min with 4% paraformaldehyde, washed in phosphate buffered saline (PBS), permeabilized with 0.1% Triton X 100, blocked with 3% bovine serum albumin, and stained with an antibody that recognizes pimonidazole adducts conjugated to DylightTM 549 fluorophore (1:200 dilution). Nuclei were stained with 4′,6 diamidino 2 phenylindole (DAPI; Invitrogen, MA, USA). Cells were visualized with a Zeiss LSM 800 confocal microscope.

### Hypoxia and ROS Detection in live cells

After 24 h of coverslip treatment, we evaluated simultaneously the levels of hypoxia and ROS in HacaT (parental and E5/E6/E7–18) live cells. At 21 h of coverslip treatment, we added both BioTrackerTM 520 Green Hypoxia Dye (Millipore, CA, USA, 5 μM final concentration) and ROS Detection Reagent Deep Red (Sigma, MO, USA, 1:1000 dilution from stock solution) through the central hole in the coverslip, or to control cells without coverslips, and after 3 h (completing 24 h of coverslip treatment) cells were visualized by confocal microscopy, and the temperature during measurements was kept at 37 °C.

### Mitochondrial membrane potential detection in live cells

To detect mitochondrial membrane potential in live cells, we used the far red fluorescent dye MitoTracker Deep Red FM (Thermo Scientific, USA) that stains mitochondria in live cells and shows accumulation which is dependent upon mitochondrial membrane potential^26,27^.

After 21 h of coverslip treatment, MitoTracker Deep Red was added through the central hole in the coverslip or to cells without coverslip (500 nM final concentration), simultaneously with BioTrackerTM 520 Green Hypoxia Dye (5 μM final concentration) and cells were incubated for another 3 h at 37 °C, and then were visualized by confocal microscopy.

### Measurements of O_2_, CO_2_, lactate and pH

HaCaT (parental and E5/E6/E7–18) cells were incubated with or without coverslips for 24 h. After this period, cells supernatants were collected and analyzed immediately in a ABL800Flex radiometer to obtain values of pH, O_2_, lactate and CO_2_ levels as well as levels of important extracellular ions (see Appendix 1).

### Oxygen consumption rate (OCR) and extracellular acidification rate (ECAR) measurements

OCR and ECAR were measured in HaCaT (parental and E5/E6/E7–18) cell using a Seahorse XF24 Extracellular Flux Analyzer (Agilent, USA). Seahorse XF Wave Software was used to analyze the data.

### Statistical analysis

Statistical analysis and graphical presentation were conducted using GraphPad Prism version 8.0.1 (244) software (GraphPad Software Inc., San Diego, CA, USA). All experiments were performed in triplicate and data were presented as the mean ± standard deviation (SD). Data were analyzed by One-Way unpaired ANOVA followed by Tukey’s HSD post-hoc test with the exception of data retrieved from Seahorse Analyzer, which was analyzed using Student`s Test in order to compare only two means.

## Supplementary Information

Supplementary Information.

## Data Availability

All data associated with this study are present in the paper or the Supplementary Materials.
