# Author Correction: Hypoxia, acidification and oxidative stress in cells cultured at large distances from an oxygen source

**DOI:** 10.1038/s41598-023-36156-7

**Published:** 2023-06-21

**Authors:** Natali D’Aiuto, Jimena Hochmann, Magdalena Millán, Andrés Di Paolo, Ronell Bologna-Molina, José Sotelo Silveira, Miguel Arocena

**Affiliations:** 1 Cátedra de Bioquímica y Biofísica, Facultad de Odontología, Universidad de la República, Montevideo, Uruguay; 2 Departamento de Genómica, Instituto de Investigaciones Biológicas Clemente Estable, Montevideo, Uruguay; 3 Departamento de Fisiología, Facultad de Medicina, Universidad de la República, Montevideo, Uruguay; 4 Departamento de Patología Molecular, Facultad de Odontología, Universidad de la República, Montevideo, Uruguay; 5 Sección Biología Celular, Facultad de Ciencias, Universidad de la República, Montevideo, Uruguay

Correction to: *Scientific Reports* 10.1038/s41598-022-26205-y, published online 15 December 2022

The Acknowledgements section in the original version of this Article was incomplete.

“This work was funded by Comisión Sectorial de Investigación Científica (CSIC), Programa de Desarrollo de las Ciencias Básicas (PEDECIBA) and Agencia Nacional de Investigación e Innovación (ANII). We are grateful to Ines Marmisolle for her assistance in mitochondrial membrane potential experiments. We are also grateful to Valeria Valez and Martín Angulo for assistance with the Seahorse XF24 Extracellular Flux Analyzer and the ABL800Flex radiometer, respectively.”

now reads:

“This work was funded by Comisión Sectorial de Investigación Científica (CSIC), Programa de Desarrollo de las Ciencias Básicas (PEDECIBA) and Agencia Nacional de Investigación e Innovación (ANII). We are grateful to Ines Marmisolle (Centro de Investigaciones Biomédicas, CEINBIO, Facultad de Medicina, UdelaR) for her assistance in mitochondrial membrane potential experiments. We are also grateful to Valeria Valez (Centro de Investigaciones Biomédicas, CEINBIO, Facultad de Medicina, UdelaR) and Martín Angulo (Departamento de Fisiopatología, Hospital de Clínicas, Facultad de Medicina, UdelaR) for assistance with the Seahorse XF24 Extracellular Flux Analyzer and the ABL800Flex radiometer, respectively.”

The original Article has been corrected.

