# Supplementary material for: Hypoxia, acidification and oxidative stress in cells cultured at large distances from an oxygen source
Source: Sci Rep. 2022 Dec 15;12:21699. doi: 10.1038/s41598-022-26205-y (PMC9755289; doi:10.1038/s41598-022-26205-y)
Supplement: Supplementary file 1 — Supplementary Information. [file 41598_2022_26205_MOESM1_ESM.docx]

**Appendix I: pH dependence on pCO_2_ and lactate**

To derive a simplified expression that relates pH to the CO_2_ produced by respiration (with concomitant carbonic acid and bicarbonate production) and the lactate produced by anaerobic glycolysis, we have to consider first the relation between pCO_2_ and bicarbonate, given by the Kassirer-Bleich approximation (Kassirer and Bleich, 1965):

$\left[ {HCO}_{3}^{\text{-}} \right]=\frac{\text{K}_{a}*\text{k}_{H}*\text{pCO}_{2}}{\left[ H^{\text{+}} \right]}$ (1)

where K_a_ is the dissociation constant for carbonic acid, and k_H_ is the constant that relates the concentration of carbonic acid to pCO_2_. K_a_ is equal to 10^-6.1^ in mol/L. k_H_ is approximately 0.03 (mmol/L)/mmHg. Therefore, converting K_a_ to mmol/L and substituting in (1), we obtain:

$\left[ {HCO}_{3}^{\text{-}} \right]\approx\frac{\text{0.000024}*\text{pCO}_{2}}{\left[ H^{\text{+}} \right]}$ (2)

The number "0.000024" is the product of K_a_ (expressed in mmol/L) and k_H_, and therefore its units are (mmol/L)^2^/mmHg. Next, we have to consider the dissociation of lactic acid to lactate. The pKa for lactic acid is 3.86, and therefore at ranges of pH around 7 we can assume that protonation of lactate is negligible. Finally, we have to consider electrical neutrality. In addition to bicarbonate, important ions that have to be taken into account for electrical neutrality are Na^+^, Cl^-^, K^+^ and Ca^2+^ (Morgan, 2009), for which the concentration in the medium is measured by the radiometer ABL800 Flex (see Material and Methods). We do not consider the contribution of other inorganic ions, such as Mg^2+^, SO_4_^2-^ or PO_4_^3-^, which are present in the culture medium at concentrations inferior to 1 mmol/L (for comparison, Na^+^ and Cl^-^ are in the range of 120-160 mmol/L, approximately). Regarding organic ions, we make the assumption that lactate is the main organic ion, with concentrations measured in our experiments in the range of approximately 7-12 mmol/L, whereas for instance pyruvate is present in DMEM culture medium at approximately 1 mmol/L. Moreover, at pH values near 7, the contribution of H^+^ and OH^-^ is negligible. Therefore, the equation for electrical neutrality can be written as:

$\left[ {Na}^{\text{+}} \right]+\left[ K^{\text{+}} \right]+2*\left[ {Ca}^{\text{+}} \right]=\left[ {HCO}_{3}^{\text{-}} \right]+\left[ lactate \right]+\left[ {Cl}^{\text{-}} \right]$ (3)

Replacing [HCO_3_^-^] by equation (2) and rearranging, we obtain:

$\left[ H^{\text{+}} \right]\approx\frac{0.000024*{pCO}_{2}}{\left( \left[ {Na}^{\text{+}} \right]+\left[ K^{\text{+}} \right]+2*\left[ {Ca}^{\text{+}} \right]-\left[ {Cl}^{\text{-}} \right] \right)-\left[ \text{lactate} \right]}$ (4)

Defining the term ([Na^+^]+[K^+^]+2*[Ca^+^]-[Cl^-^]) as Δ_inorganic ions_, we obtain the final expression for [H^+^] in mmol/L as a function of pCO_2_, lactate and a term containing the difference in charge between the main ions in the medium:

$\left[ H^{\text{+}} \right]\approx\frac{0.000024*{pCO}_{2}}{\Delta_{inorganicions}-\left[ \text{lactate} \right]}$ (5)

Next, we can introduce our experimental data in equation (5), to assess whether it predicts accurately the experimental pH values obtained. For instance, in one of our measurements of the conditioned medium of HaCaT E5/E6/E7-18 cells in control conditions, we obtained: pCO_2_ = 24.9 mmHg, [lactate] = 9.4 mmol/L and Δ_inorganic ions_ = 37.36 mmol/L. The experimental pH value was 7.75. Replacing the values for pCO_2_, lactate and Δ_inorganic ions_ in equation (5), we obtain: [H^+^] = 0.0000214 mmol/L, corresponding to a pH value of 7.67. That is, we find a difference of 0.08 pH units between the experimental and the predicted pH value.

As another example, we can consider one of our measurements of the conditioned medium of HaCaT E5/E6/E7-18 cells in conditions of coverslip induced hypoxia, where we obtained: pCO_2_ = 49.6 mmHg, [lactate] = 12 mmol/L and Δ_inorganic ions_ = 37.52 mmol/L. The experimental pH value was 7.31. Using equation (5), we obtain: [H^+^] = 0.0000466 mmol/L, corresponding to a pH value of 7.33. That is, we find a difference of 0.02 pH units between the experimental and the predicted pH value. For all of our experimental values, we have found differences ranging between 0.01 and 0.2 pH units between the experimental pH values and the pH values predicted by equation (5), indicating that this expression accurately predicts pH values in our experimental conditions.

Next, we can use equation (5) to estimate the relative importance of pCO_2_ and lactate increase in the acidification observed in conditions of coverslip induced hypoxia. To do this, we can vary pCO_2_ and lactate separately. For example, we can ask what will be the pH value if pCO_2_ is maintained in its control condition value, while lactate increases to its value in the coverslip induced hypoxia condition. Considering the two examples mentioned, this would correspond to pCO_2_ = 24.9 mmHg and [lactate] = 12 mmol/L. Using the Δ_inorganic ions_ value from the control condition and applying equation (5), we obtain a predicted pH value of 7.63. That is, the pH would decrease from a predicted value in control conditions of 7.67 to a predicted value of 7.63 in hypothetical conditions where only lactate is allowed to increase. However, if instead lactate is kept at its control condition value and only pCO_2_ is allowed to increase to its value in the coverslip induced hypoxia condition (49.6 mmHg in the previous example), we obtain a predicted pH value of 7.37, indicating a much larger decrease in pH. Therefore, we can conclude from this analysis that, in our experimental conditions, the measured increase in pCO_2_ has a much greater role than the measured increase in lactate in causing the acidification observed during coverslip induced hypoxia.

**References**

Kassirer, J. P., & Bleich, H. L. (1965). Rapid estimation of plasma carbon dioxide tension from pH and total carbon dioxide content. New England Journal of Medicine, 272(20), 1067-1068.

T.J. Morgan, The Stewart approach--one clinician’s perspective., Clin. Biochem. Rev. 30 (2009) 41–54.
